# Distinct genetic programs guide *Drosophila* circular and longitudinal visceral myoblast fusion

**DOI:** 10.1186/1471-2121-15-27

**Published:** 2014-07-08

**Authors:** Anja Rudolf, Detlev Buttgereit, Matthias Jacobs, Georg Wolfstetter, Dörthe Kesper, Michael Pütz, Susanne Berger, Renate Renkawitz-Pohl, Anne Holz, Susanne F Önel

**Affiliations:** 1 Developmental Biology, Department of Biology, Philipps-Universität Marburg, Karl-von-Frisch-Straße 8, Marburg 35043, Germany; 2 Present address: INSERM U1016 Institut Cochin, Département Génétique et Développement, 24 Rue du Faubourg Saint Jacques, Paris 75014, France; 3 Institut für Allgemeine Zoologie und Entwicklungsbiologie, Justus-Liebig-Universität Gießen, Stephanstraße 24, Giessen 35390, Germany; 4 Present address: Department of Molecular Biology, Building 6 L, Umeå University, Umeå 90187, Sweden; 5 Present address: Institute of Pathobiochemistry and Molecular Diagnostics, Philipps-Universität Marburg, Hans-Meerwein-Straße 2, Marburg 35043, Germany; 6 Present address: Institute for Neurology, Clinical Neurobiology, FB20 Philipps-Universität Marburg, Baldingerstraße, Marburg 35043, Germany; 7 Present address: Life and Medical Sciences Institute, Universität Bonn, Carl-Troll-Straße 31, Bonn 53115, Germany

**Keywords:** Visceral musculature, Actin regulation, FuRMAS, Myoblast fusion, Site-specific mRNA localization, Differential transcriptional control, Scar/Wave, Blow, Rols, Kette/Nap-1

## Abstract

**Background:**

The visceral musculature of *Drosophila* larvae comprises circular visceral muscles tightly interwoven with longitudinal visceral muscles. During myogenesis, the circular muscles arise by one-to-one fusion of a circular visceral founder cell (FC) with a visceral fusion-competent myoblast (FCM) from the trunk visceral mesoderm, and longitudinal muscles arise from FCs of the caudal visceral mesoderm. Longitudinal FCs migrate anteriorly under guidance of fibroblast growth factors during embryogenesis; it is proposed that they fuse with FCMs from the trunk visceral mesoderm to give rise to syncytia containing up to six nuclei.

**Results:**

Using fluorescence in situ hybridization and immunochemical analyses, we investigated whether these fusion events during migration use the same molecular repertoire and cellular components as fusion-restricted myogenic adhesive structure (FuRMAS), the adhesive signaling center that mediates myoblast fusion in the somatic mesoderm. Longitudinal muscles were formed by the fusion of one FC with Sns-positive FCMs, and defects in FCM specification led to defects in longitudinal muscle formation. At the fusion sites, Duf/Kirre and the adaptor protein Rols7 accumulated in longitudinal FCs, and Blow and F-actin accumulated in FCMs. The accumulation of these four proteins at the fusion sites argues for FuRMAS-like adhesion and signaling centers. Longitudinal fusion was disturbed in *rols* and *blow* single*,* and *scar wip* double mutants. Mutants of *wasp* or its interaction partner *wip* had no defects in longitudinal fusion.

**Conclusions:**

Our results indicated that all embryonic fusion events depend on the same cell-adhesion molecules, but that the need for Rols7 and regulators of F-actin distinctly differs. Rols7 was required for longitudinal visceral and somatic myoblast fusion but not for circular visceral fusion. Importantly, longitudinal fusion depended on Kette and SCAR/Wave but was independent of WASp-dependent Arp2/3 activation. Thus, the complexity of the players involved in muscle formation increases from binucleated circular muscles to longitudinal visceral muscles to somatic muscles.

## Background

The body wall musculature of *Drosophila melanogaster* arises during embryogenesis and metamorphosis by fusion within the somatic mesoderm of two cell types: the founder cells (FCs) and the fusion-competent myoblasts (FCMs). This fusion generates syncytial myotubes that allow movement of larvae and adults (reviewed in [1,2]). Interestingly, the visceral muscles surrounding the midgut and hindgut are also syncytial. This visceral musculature forms a web-shaped syncytium around the gut and is comprised of binucleated circular muscle fibers interwoven with multinucleated longitudinal muscles. Correct establishment of the circular and longitudinal muscles is a prerequisite for subsequent gut development, since they constrict the gut into four chambers at the end of embryogenesis [3,4]. Both muscle types persist during metamorphosis [5-10].

The circular visceral muscles in *Drosophila* arise by fusion of one circular visceral FC with one visceral FCM, both from the trunk visceral mesoderm [8,11]. While it has been proposed that a common pool of visceral FCMs exist, the circular and longitudinal FCs are distinct and originate from different mesodermal primordia. Both the circular FCs and the visceral FCMs originate from the trunk visceral mesoderm (TVM), which is characterized by expression of the homeodomain transcription factor Bagpipe (Bap) and the FoxF factor Biniou [12-14]. The fate of circular FC is determined by both Delta/Notch signaling and Ras/MAPK signaling via the receptor tyrosine kinase ALK and its ligand Jelly Belly [15-19]. These FCs fuse one-to-one with a visceral FCM. Rather than producing massive syncytia, this fusion results in syncytia interconnected with multiple cytoplasmic bridges. The binucleate cells stretch until they enclose the whole gut [8,10,11].

In contrast to the circular visceral muscles, the longitudinal muscles contain up to six nuclei, and it is therefore thought that they develop through several fusion events. The longitudinal FCs originate from the caudal visceral mesoderm. These cells are characterized by the basic helix-loop-helix transcription factor HLH54F, and after one mitotic division, they migrate anteriorly along the trunk visceral mesoderm (TVM) under the control of fibroblast growth factor receptor signaling [4,20-24]. It is proposed that once these longitudinal FCs reach the TVM, they fuse with the remaining visceral FCMs after circular fusion is completed [8,19].

In somatic myoblast fusion, numerous cell-adhesion and intracellular molecules are essential, e.g., the immunoglobulin superfamily members Dumbfounded/Kin of Irre (Duf/Kirre), Roughest/Irregular chiasm C (Rst/IrreC), and Sticks and Stones (Sns) ([25-27]); the adaptor molecule Rolling pebbles (Rols), which interacts with the intracellular domain of Duf/Kirre [28]; and proteins involved in Arp2/3-dependent actin polymerization, e.g., Blown fuse (Blow), Kette, Myoblast city (Mbc), the Wiskott-Aldrich-syndrome protein (WASp)-interacting protein Wip [also known as Verprolin1 (Vrp1) and Solitary (Sltr)], and the Arp2/3 nucleation-promoting factors (NPFs) Scar/Wave and WASp (reviewed recently by [1,29]). Upon cell adhesion, Arp2/3-dependent F-actin formation is involved in the formation of a dense F-actin focus in somatic FCMs and a thin F-actin sheath in somatic FCs [30].

During somatic myoblast fusion, the FCM-specific protein Blow overlaps with the F-actin foci in FCMs at cell–cell contact points and stabilizes the WASp/Wip complex located there [31-33]. Kette is a component of the regulatory Scar/Wave complex and is expressed in somatic FCs and FCMs [34,35], and is required during the second fusion phase. Kette and Scar/Wave both mediate myoblast fusion in FCs and act together with Wip and WASp in FCMs [34-37]. Recent findings suggest that *kette, wasp,* and *wip* mutants can still form binucleate circular muscles [38]. Therefore, it has been proposed that Arp2/3-mediated actin polymerization is not essential for visceral myoblast fusion.

It is still not known whether proteins involved in somatic myotube formation are essential for the fusion events that occur after the longitudinal FCs arrive at the TVM. In FCs of the somatic mesoderm, Duf/Kirre and Rols7 localize as part of the fusion-restricted myogenic adhesive structure (FuRMAS), which is postulated to be an essential signaling center at sites of cell contact. Rols7 is required to complete the second fusion phase as an adaptor protein that links cell recognition and adhesion via Duf/Kirre. Rols7 possibly remodels actin or is involved in widening of the adhesion ring within the FuRMAS; it is proposed that the widening of the ring triggers myoblast fusion [32,39-43].

Here we present evidence that the longitudinal muscles arise by fusion of the longitudinal FCs with Sns-positive FCMs during FC migration. We showed that FuRMAS-like structures containing Duf/Kirre, Rols7, Blow, and F-actin exist in longitudinal visceral muscles at the site of fusion. These structures were smaller than those found during somatic myogenesis. Also Blow, Kette, and Scar/Wave were required for this event. However, we found no evidence for WASp/Wip-activated Arp2/3-dependent longitudinal visceral myoblast fusion, which is characteristic for somatic myoblast fusion [32,36]. These observations suggested that the molecular players of myoblast fusion increase in complexity from the process of forming binucleated circular visceral muscles to the process of forming small syncytial longitudinal visceral muscles to the process of forming somatic body wall muscles.

## Material and methods

### *Drosophila* stocks

The following *Drosophila* stocks were used in this study: *Df(3 L)BK9/TM3,Sb,Dfd-lacZ* (Deficiency for *rols7,* BDSC)*, rols*^*XX117*^*/TM3,Sb,Dfd-lacZ*[43], *blow*^*1*^/*CyO* and *blow*^*2*^/*CyO*[44], *kette*^*J4–48*^*/TM3,Sb,Dfd-lacZ* and *kette*^*G1–37*^/*TM3,Sb,Dfd-lacZ*[45], *mbc*^*C1*^/*TM3,Sb* and *mbc*^*D11.2*^*/TM3,Ser*[25], *lmd*^*202*^/*TM3,Sb,Dfd-lacZ* (Holz and Renkawitz-Pohl, unpublished), *rp298-lacZ*[46], *sns-mCherry-NLS*[47], *bap-lacZ*[12], *HLH54F-lacZ*[21], *HLH54F-GFP*[24], *twist promoter-GFP-actin*[35], *rolsIn1-lacZ* (see below), the protein trap lines *P{PTT-un1}sls*^*ZCL2144*^ (*sls*::*GFP*) [48] and *P{unk}trol*^*GFP311*^ (*trol*::*GFP*) [49], *wip*^*D30*^*/CyO*[50], *wip*^*f06715*^*/CyO,hg-lacZ*, *wip*^*f06715*^*scar*^*Δ37*^*/CyO,hg-lacZ, arp3*^*schwächling*^*wasp*^*3D3–035*^*/TM3,Sb,Dfd-lacZ*[36], and *arp3*^*schwächling*^/*TM3,Sb,Dfd-lacZ*[36]. The *scar*^*Δ37*^ allele was obtained from the Bloomington *Drosophila* Stock Center. To distinguish between homozygous mutants and heterozygous flies, balancer chromosomes carrying *lacZ* or *GFP* insertion markers (Bloomington stocks 6662 and 6663) were used. As the wild-type reference, we used *w*^*1118*^ or balanced sibling embryos.

### Immunohistochemical analyses of *Drosophila* embryos

Embryos were fixed and immunohistochemically analyzed as described previously [32]. Guts were prepared from embryos and larvae fixed with 4% formaldehyde and not treated with methanol and were stained the same way as whole mount embryos. The following antibodies were used at the noted dilutions: mouse anti-FasIII [51] 1:50 (Developmental Studies Hybridoma Bank), rabbit anti-Duf/Kirre [28] 1:1000, rabbit anti-β3Tub [52] 1:3000, rabbit anti-β-gal 1:5000 (Biotrend), rabbit anti-Blow [10] 1:200, rabbit anti-DMef2 [53] 1:500, rabbit anti-GFP 1:500 (abcam), rabbit anti-Rols7 (directed against the first 300 amino acids of Rols7) 1:500, rat anti-RFP (Clontech) 1:1000, and rat anti-Tm (abcam) 1:1000. Embryos stained with anti-Rols7, anti-Duf/Kirre, and anti-Blow were heat fixed, and the reaction was enhanced using an Individual Indirect Tyramide Reagent Pack (Perkin Elmer). Primary antibodies were detected using either biotinylated secondary antibodies at a dilution of 1:250 and the Vectastain Elite ABC Kit (Vector Laboratories) or fluorescent-labeled antibodies at a dilution of 1:200 (Dianova). DNA was stained with Hoechst reagent (5 μg/ml; Sigma-Aldrich), and F-actin was stained with TRITC-coupled phalloidin (10 μg/ml; Sigma-Aldrich). For all stainings, specimens were embedded in Epon or Fluoromount-G™ (Southern Biotech) and observed under a Zeiss Axiophot 2 microscope, Zeiss AxioObserver.Z1 inverse microscope, or Leica TCS Sp2 confocal microscope.

### Fluorescence *in situ* hybridization (FISH)

A DIG-labeled *rols* probe directed at the transcript encoding the C-terminus of Rols7 was synthesized by *in vitro* transcription of *rols* cDNA LD1 [43] using a DIG-RNA labeling kit (Roche Diagnostics) and following the manufacturer’s instructions. Formaldehyde-fixed embryos were hybridized *in situ* with the DIG-labeled *rols* probe essentially as described in Lécuyer et al. [54]. DIG-labeled *rols* probes bound to embryos were detected with biotinylated anti-DIG antibody (1:2000; Roche Diagnostics) and the TSA™ Fluorescein System (Perkin Elmer). Fluorescence *in situ* hybridizations were analyzed by confocal microscopy at various depths within the samples (stacks), which ensured that many ends of the spindle-like FCs were visible in a single picture.

### Generation of transgenic flies carrying promoter constructs

*rols7* and *rols6* reporters were constructed by ligating different parts of the presumptive control regions of the genes to the *Escherichia coli lacZ* gene and integrating these constructs into pChap to establish transgenic *Drosophila* lines as reported previously [55]. In addition, the nucleotides sequence encoding the first intron of the *rols* gene was cloned into the vector pChabHsp43-lacZ [56], yielding *rolsIn1-lacZ,* which was injected into *w*^*1118*^ flies to establish transgenic lines following standard procedures.

**Figure 1:**
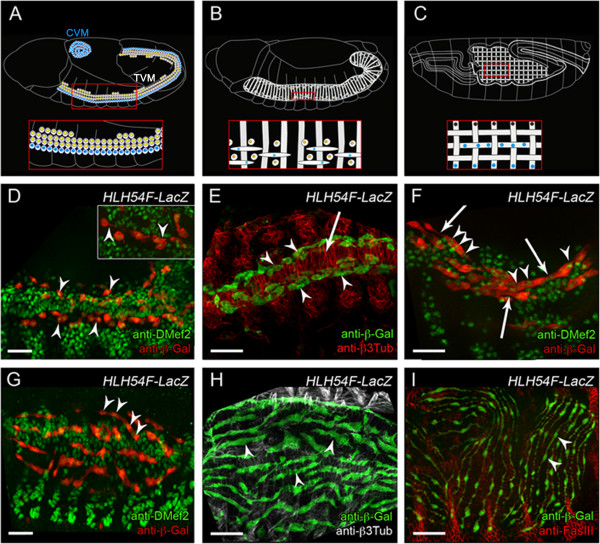
**Longitudinal FC migration and fusion lead to the formation of longitudinal visceral muscles. (A–C)** Schematic representation of the development of the visceral musculature surrounding the midgut. FCMs have yellow nuclei, and FCs have blue nuclei. Lateral views of the mature visceral muscles around the midgut are shown. **(A)** At late stage 11, the visceral FCMs are localized in the trunk mesoderm (TCM), circular visceral FCs are organized as a layer adjacent to visceral FCMs, and longitudinal visceral FCs localize in the caudal mesoderm (CVM). **(B)** Early stage 12 embryo, with binucleated visceral muscles (gray stripes). Many FCMs are localized near the binucleated visceral muscles, and longitudinal spindle-shaped mononucleated FCs are migrating over the layer of visceral circular muscles. **(C)** Stage 16 embryo with a network of circular and longitudinal visceral muscles. All nuclei of longitudinal visceral muscles express the rP298 enhancer (blue); one nucleus of the circular visceral muscles is rP298 positive (blue), and the other is rP298 negative (brown). **(D–I)** Embryos with longitudinal FCs expressing *HLH54F-lacZ.* Nuclei of mesodermal cells in **(D, F)** were visualized by anti-DMef2 staining. **(D)** Mononucleated, migrating longitudinal FCs (arrowheads) in early stage 12 embryos. Inset: magnification showing cells contacting each other (arrowhead). **(E)** Longitudinal FCs (arrowheads) arranged along the stretching, β3Tub-expressing circular muscles (arrow) in stage 12 embryos. **(F)** Late stage 12 embryo with multinucleated longitudinal FCs. Arrowheads point to nuclei of binucleated and trinucleated cells; arrows indicate cell contacts. **(G, H)** Stage 14 embryos: at the time when the circular muscles stretched, the multinucleated longitudinal FCs stretched perpendicularly. Arrowheads in **(G)** point to nuclei of one multinucleated cell; arrowheads in **(H)** point to cells stretching in anterior–posterior directions. **(I)** Embryo at the end of development. Longitudinal muscles cover the gut evenly. Arrowheads indicate nuclei of multinucleated muscle. Scale bars: 20 μm.

## Results

### Longitudinal and circular visceral muscles differ from somatic muscles in several aspects

The gut musculature of *Drosophila* larvae is multinuclear and striated [6-8] and comprised of a dense network of circular and longitudinal muscles that can be visualized by scanning electron microscopy [10]. At the light microscopy level, the network can be visualized with the protein-trap allele *sls*::*GFP*[48,49,57-59], the ECM protein Trol/Perlecan tagged internally with GFP *(trol*::*GFP)*[49,60], and TRITC-coupled phalloidin to visualize F-actin (Additional file 1: Figure S1A–C). Trol/Perlecan is an ECM component; the ECM around the trunk visceral mesoderm (TVM) is required for longitudinal FCs to migrate along the TVM [61,62].

The morphological data are schematically summarized in Figure 1A–C. At late stage 11, the trunk mesoderm contains on each side of the embryo a row of FCs for the circular visceral muscles and two to three rows of FCMs, which have been proposed to be a common pool for circular visceral muscles and longitudinal visceral muscles. The FCs for longitudinal visceral muscles are determined in the caudal region of the embryo (Figure 1A). At early stage 12 (Figure 1B), the circular visceral muscles are binuclear and arose by one-to-one fusion of a circular FC from the TVM with a visceral FCM [8,11]. In *rp298-lacZ*[46], the FC-derived nucleus in the small syncytia remained β-Gal positive, while the nucleus derived from the visceral FCM was β-Gal negative (Figure 1C and Additional file 1: Figure S1C; [11]), as observed earlier in embryos at stage 12. The binucleated circular visceral muscles stretch out in the dorsoventral direction. The FCs of the longitudinal visceral muscles migrate over the stretching circular visceral muscles and reach the anterior part of the trunk mesoderm in early stage 12. At this time, the longitudinal visceral muscles are still mononucleated (Figure 1D) and are surrounded by FCMs (Figure 1B). What happens between this stage and late embryonic stages, with their typical network of circular and longitudinal visceral muscles, is still unknown (Figure 1C).

**Figure 2:**
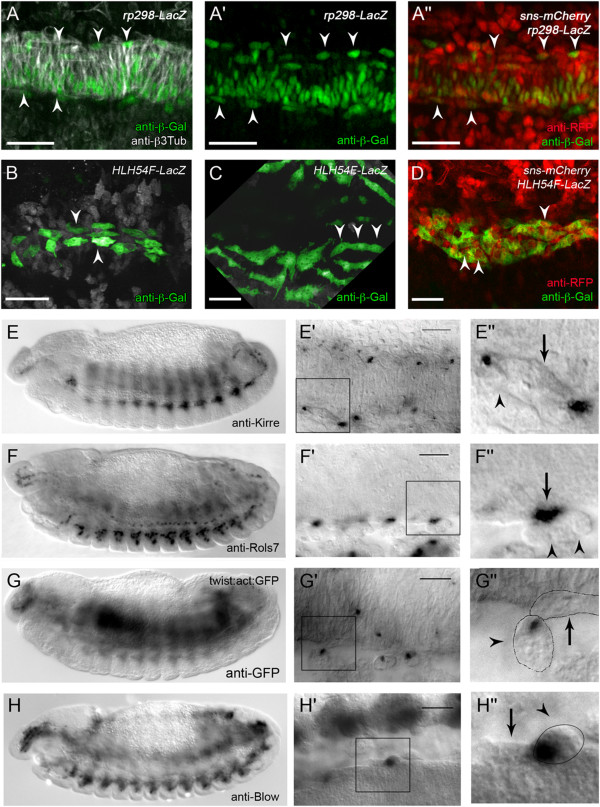
**Duf/Kirre, Rols, F-actin, and Blow are expressed in foci during fusion of longitudinal FCs with FCMs. (A–A**”**)** Embryo expressing *rp298-lacZ* in somatic and visceral FCs. **(A)** Mesodermal cells visualized with anti-β3-Tubulin. Arrowheads point to β-Gal-positive longitudinal FCs. **(A**’**)** Longitudinal FCs (arrowheads) are mononucleated at this stage. **(A**”**)** Embryo also expressing *sns-NLSmCherry* in somatic and visceral FCMs. **(B–D)** Embryos expressing *HLH54F-lacZ*. **(B)** Early stage 12 embryo; longitudinal FCs (arrowheads) still appear to be mononucleated. **(C)** Embryo in later stage 12, with multinucleated syncytia (arrowheads). **(D)** Embryo also expressing *sns-mCherry*; some syncytia appear to be sns-mCherry positive (arrowheads). **(E–H**”**)** Histochemical staining of wild-type embryos at mid or late stage 13. The embryos are shown as an overview **(E, F, G, H)** and at two magnifications focusing on longitudinal myogenesis **(E**’**, F**’**, G**’**, H**’ **and E**”**, F**”**, G**”**, H**”**)**. Either heat-fixed embryos **(E, F, H)** or formaldehyde fixed embryos **(G)** were stained with anti-Kirre **(E–E**”**)**, anti-Rols7 **(F–F**”**)**, anti-GFP **(G–G**”**)**, or anti-Blow **(H–H**”**)** antibodies. Arrows point to longitudinal FCs/growing myotubes; arrowheads indicate FCMs. At higher magnifications, local concentrations of the proteins are visible either on the side of the FC/elongation myotube, as in the case of anti-Kirre and anti-Rols7 **(E**’**, E**”**; F**’**, F**”**)**, or in the FCM at the site of attachment for *twi*::*act*::*GFP***(G**’**, G**”**)** and anti-Blow **(H**’**, H**”**)**. Scale bars: 20 μm.

### The longitudinal visceral FCs form multinucleated nascent myotubes during migration and stretch to thin long myotubes at the end of embryogenesis

Since far less is known about myoblast fusion during development of longitudinal visceral muscles than of circular visceral muscles, we focused on longitudinal muscles. We used flies carrying the reporter construct *HLH54F-lacZ,* in which longitudinal FCs are marked by β-Gal expression [21]. These cells migrate from the circular visceral muscles anteriorly along the TVM from stage 11 until stage 13 [20-22,24]. When we focused on the time and efficiency of myoblast fusion in these migrating cells, we observed that they were mononucleated when they arrived at the TVM in early stage 12 of development (Figure 1D). At this time point, they formed protrusions, and in some cases, we observed closely adjacent *HLH54F-lacZ-*positive cells (Figure 1D insert, arrowheads). The longitudinal FCs were arranged dorsally and ventrally of the TVM when they migrated (Figure 1E). At late stage 12, we detected binucleated and trinucleated nascent myotubes, in part connected by thin cytoplasmic bridges (Figure 1F, arrows). By the time the circular muscles had stretched dorsally, the longitudinal FCs were arranged perpendicularly to the circular muscles and already contained several nuclei (Figure 1G and H, arrowheads). At the end of embryogenesis, when the gut was constricted, the longitudinal gut muscles covered the whole midgut evenly. At this stage, β-Gal expression in myotubes appeared in very thin areas, not much wider than the nuclei, with even thinner protrusions between them (Figure 1I).

### During longitudinal visceral muscle myogenesis, Duf/Kirre, Rols7, and Blow localize at distinct foci at the sites of fusion

We further analyzed the fusion process of longitudinal FCs and visceral FCMs by using cell-type-specific *duf*- and *sns*-reporter constructs. The adhesion molecule Duf/Kirre is expressed in all somatic and visceral FCs, while Sns is expressed in all somatic and visceral FCMs [8,11]. We observed that in embryos carrying *rp298-lacZ,* the nuclei of the longitudinal FCs were β-Gal positive at stages when fusion presumably occurred (Figure 2A–A’), concordant with earlier data that showed *rp298-lacZ-*positive nuclei in migrating longitudinal FCs as well as in the mature longitudinal visceral muscles [8,11].

**Figure 3:**
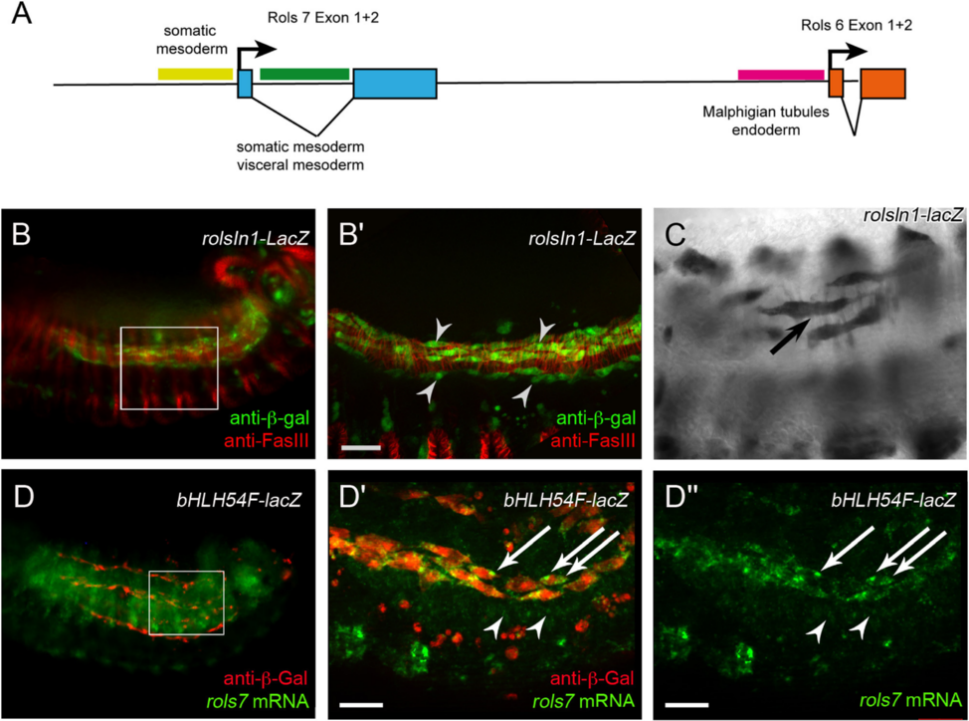
**Rols7 is transcribed in TVM and circular visceral muscles. (A)** Scheme of the *rolling pebbles* promoter region. *rols7*: yellow, 3 kb upstream region required for maximum expression in the somatic mesoderm; green, intron with control elements for transcription in the visceral mesoderm and somatic muscles; blue, exons 1 and 2 of *rols7. rols6*: pink, approximately 1.2 kb upstream region essential for expression in the endoderm and Malpighian tubules; orange, exons 1 and 2. **(B)** Expression of the *roIsIn1-lacZ* reporter construct, which contains the regulatory region between exon 1 and 2 of *rols7* (green in **A**). **(B’)** Magnification of boxed area in **(B)**; β-Gal (green fluorescence) in longitudinal FCs (arrowheads) along the TVM, marked by anti-FasIII (red fluorescence). **(C)** β-Gal-positive stretching circular muscles (arrow). **(D)***In situ* hybridization of *bHLH54F-lacZ* embryos using a *rols7* probe (green fluorescence). Longitudinal visceral FCs were stained with anti-β-Gal (red fluorescence). **(D’ and D”)** Magnification of C: arrowheads, *rols7* mRNA in β-Gal-negative circular visceral FCs; arrows, *rols7* mRNA in β-Gal-positive longitudinal FCs. Scale bars: 20 μm.

To follow the visceral FCMs, we established flies carrying both *sns-NLSmCherry*[47] and *rp298-lacZ*. At stages when the longitudinal FCs migrated and were still mononucleated, *sns-NLSmCherry*-positive FCMs from the somatic mesoderm and the TVM were in proximity to the rp298-positive FCs (Figure 2A”, arrowheads).

To follow the fate of *sns-mCherry*-expressing FCMs, we established a fly strain carrying *sns-NLSmCherry* (nuclear signal) and *HLH54F-lacZ* (cytoplasmic signal). *HLH54F-lacZ* also allowed us to follow the nascent longitudinal muscles, which increase in size during development (Figure 2B–D). These nascent myotubes were still surrounded by numerous *sns-NLSmCherry*-expressing myoblasts (Figure 3D). The *sns*-NLSmCherry signal often appeared to be in the nuclei of the nascent longitudinal myoblasts, which might indicate fusion between FCs and FCMs (Figure 2D, arrowheads); however, due to the numerous *sns-NLSmCherry-*positive cells in proximity to these nascent myotubes, this was difficult to evaluate. Nevertheless, we hypothesized that the longitudinal FCs fuse with *sns-NLS mCherry*-positive visceral FCMs.

Consequently, we analyzed the localization of fusion-relevant proteins during longitudinal myogenesis in mid and late stage 13, when fusion takes place to create the longitudinal visceral muscles (see Additional file 2: Figure S2 for circular visceral myogenesis). First, we analyzed whether and where Duf/Kirre and Rols7 are present in the longitudinal visceral FCs (Figure 2E–E”, F–F”). Duf/Kirre was expressed in the longitudinal FCs while they migrated over the circular visceral muscles, in agreement with the expression of *rp298-lacZ* (compare Figure 2E’ to Figure 2A’). Duf/Kirre often localized with a striking subcellular distribution at both ends of the spindle-like FCs (Figure 2E’, arrow). At a higher magnification, it became evident that Duf/Kirre was limited to those sites of the FC that were in contact with a visceral FCM (Figure 2E”, arrowhead). Next, we used an antibody directed against the first 300 amino acids of Rols7 and detected the protein in the visceral mesoderm (Figure 2F). In part, Rols7 was found in foci at the ends of the spindle-like longitudinal FCs (Figure 2F’). At higher magnification, it was evident that these foci were also at the contact sites with an FCM (Figure 2F”).

This accumulation of Duf/Kirre and Rols7 is comparable to the FuRMAS structure observed during fusion in the somatic mesoderm [32,42]. The FuRMASs are characterized by the ring-like distribution of Duf/Kirre, Rst/IrreC, Rols, and Sns in FCs and by an F-actin-rich core in the FCMs (for recent reviews, see [1,29]). To analyze whether F-actin foci appear in FCMs during longitudinal visceral muscle myogenesis, we investigated actin-GFP expression under the control of the *twist* promoter. Indeed, we observed F-actin foci in those FCMs (Figure 2G”, arrowhead) that contact the longitudinal FCs (Figure 2G’, G” arrow).

Blow is a regulator of WASp-mediated Arp2/3-dependent F-actin polymerization during somatic myogenesis and accumulates as dense foci in FCMs [10,31,32,34]. We found Blow in FCMs that contact the longitudinal visceral FCs (Figure 2H’, H”). At these stages, the spindle-like FCs were about 15 μm in length, while the diameter of the Blow and actin foci was mainly 0.5 to 1.0 μm.

Taken together, these findings suggest that FuRMAS-like structures exist in longitudinal FCs and FCMs. However, Duf/Kirre and Rols were not observed in ring-like structures, in contrast to Duf/Kirre and Rols in somatic FCs, but rather appeared as foci. This difference might be due to the much smaller size of the observed structures compared to those in somatic myoblasts.

### The *rols7* transcript localizes in the longitudinal FCs before fusion

The *rols* gene is regulated by two promoters, which leads to *rols7* and *rols6* transcripts with specific 5’ exons [43]. We investigated which isoform of Rols is required in longitudinal FCs. We analyzed the promoter regions responsible for transcription of *rols7* and *rols6,* focusing on longitudinal myogenesis.

Indeed, the *rols7* promoter contained distinct regulatory regions for transcription in the somatic mesoderm and visceral mesoderm (see Figure 3A for a summarizing scheme). An intron between exons 1 and 2 of *rols7* controlled transcription in the circular visceral mesoderm and during development of longitudinal muscles (Additional file 3: Figure S3, *rolsIN1-lacZ* reporter). To clarify the situation in longitudinal visceral myogenesis in more detail, we used these *rolsIn1-lacZ* transgenic embryos stained with fluorescent antibodies against β-Gal and the cell surface glycoprotein Fasciclin III (FasIII, [51]) to label the membranes of the trunk mesoderm. In embryos expressing *rolsIn1-lacZ*, the longitudinal FCs clearly expressed β-Gal when they migrated along the TVM in mid-embryogenesis (Figure 3B, B’, arrowheads). Also later, when the circular muscles stretched dorsally, β-Gal was expressed in the longitudinal FCs that were already binucleated (Figure 3C). Thus, the *rolsIN1-lacZ* reporter allowed us to follow the fusion stages during longitudinal visceral myogenesis.

Rols7 was detected at distinct foci at both ends of the spindle-like FCs in the developing longitudinal visceral muscles (Figure 2F–F”). To investigate whether this particular localization of Rols7 is regulated at the level of *rols7* mRNA localization in the longitudinal FCs at this stage, we hybridized *bHLH54F-lacZ* embryos *in situ* with fluorescent probes aimed against *rols7* mRNA and stained with antibodies against β-Gal to visualize FCs (Figure 3D–D”). In the longitudinal FCs, *rols7* transcripts were mostly concentrated in speckles, and often towards the tips of the spindle-shaped cells, which indicated a targeted distribution of *rols7* mRNA during fusion (Figure 3D’ and D”, arrows).

### Rols7 is required for fusion but not for orientation or migration of longitudinal FCs

**Figure 4:**
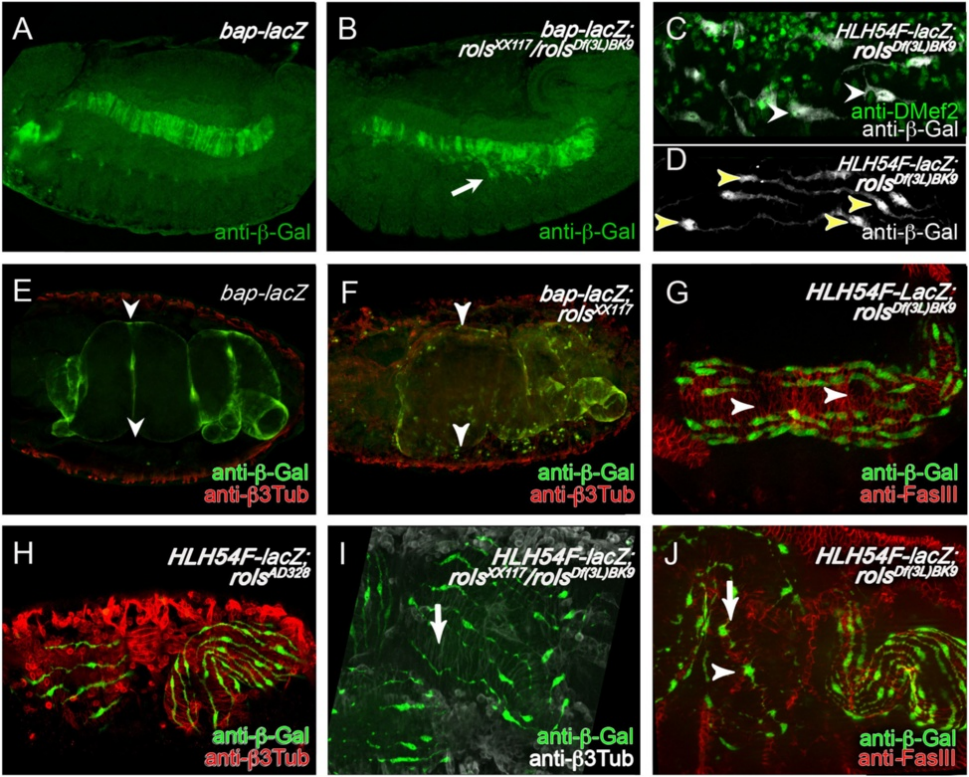
**Longitudinal muscle fusion requires *rols7*.** Expression of *bap-lacZ* (green in **A**, **B**, **E**, **F**) or *HLH54F-lacZ* (green in **G**–**J**; white in **C **and **D**) visualized by staining with fluorescent anti-β-Gal and counterstaining with anti-β3-Tubulin (anti-β3Tub red in **E**, **F**, **H**; white in **I**). Staining of visceral mesoderm with anti-Fasciclin III (anti-FasIII, red in **G** and **J**). Lateral view of wild-type embryo **(A)** and *rols* mutant embryo **(B)** at stage 12. Note the β-Gal-positive cells in the *rols* mutant embryo **(B)** along the stretching circular muscles. Dorsolateral views of stage 16 wild-type embryo **(E)** and *rols7* mutant embryo **(F)**. **(E, F)** Arrowheads point to the position of the 1^st^ midgut constriction. **(C, D)***rols7-*deficient embryos stained with anti-β-gal showing **(C)** mononucleated migrating longitudinal FCs with random protrusions (arrowheads) and **(D)** morphology of binucleated longitudinal muscles (arrowheads). **(G)** Anti-FasIII staining of *rols7-*deficient embryos in mid-embryogenesis; longitudinal FCs located dorsally and ventrally on stretching FasIII-positive circular muscles, which sometimes display small gaps (arrowheads). **(H–J)** Anterior midgut regions covered with mainly mononucleated longitudinal muscles in different *rols* alleles at the end of embryogenesis (arrowhead); posterior midgut regions with parallel-orientated longitudinal muscles. Arrows point to regions lacking longitudinal muscles.

Since *rols7* mRNA partially localized to the polar, presumptive sites of fusion in the longitudinal FCs, and since the Rols7 protein localized at distinct foci during longitudinal visceral myogenesis, we then asked whether *rols7* is required for fusion in both circular and longitudinal visceral myogenesis. We analyzed *rols*-deficient embryos expressing different reporter constructs that mark visceral FCs or FCMs. First we used *bap-lacZ* transgenic lines to distinguish between unfused visceral FCMs and unfused somatic FCMs. The transcription factor Bagpipe (Bap) is expressed in all visceral myoblasts of the trunk mesoderm. After fusion of circular FCs with neighboring FCMs to form binucleated syncytia, only a small number of remaining unfused FCMs can be detected directly beneath and above the stretching circular myotubes (Figure Three I–L in [11]). Klapper *et al.*[11] suggested that these remaining FCMs fuse with the migrating longitudinal FCs. Thus, in wild-type embryos, *bap-lacZ* mainly marks the circular visceral muscles directly after fusion (Figure 4A) and β-Gal was later expressed in the visceral muscles of the midgut (Figure 4E). In *rols7* mutant embryos, unfused β-Gal*-*positive visceral myoblasts were located in the interstitium between the somatic mesoderm and visceral mesoderm (Figure 4F).

To examine the origin of these unfused visceral myoblast cells, we analyzed *rols* mutant embryos at earlier stages of visceral muscle formation. *rols* mutants exhibited more unfused FCMs, which indicated a visceral fusion defect (compare Figure 4A and B), in agreement with expression of Rols7 in circular (Additional file 2: Figure S2B’) and longitudinal visceral FCs (Figure 2F–F”). However, *rols* mutant circular muscles stretched normally in the dorsal direction (Figure 4B and G) and the overall circular muscle morphology visible with *bap-lacZ* and other markers, such as FasIII, appeared to be mostly regular with only sporadic small gaps (Figure 4G, arrowhead). We conclude that these gaps are the result of minor failures in circular myoblast fusion and that the majority of unfused myoblasts (Figure 4F) result from failure in longitudinal visceral myotube formation.

This conclusion was supported by the midgut morphology of *rols* mutant embryos in late stages of embryogenesis. Using anti-β3-Tubulin to visualize midgut muscle morphology, we observed a chambering defect between the 1^st^ and 2^nd^ midgut chamber in *rols* mutants during late embryogenesis (Figure 4F, arrowheads). This defect resembles the midgut phenotype of known circular visceral fusion mutants [11,25], although in the *rols* mutants, the defect is most likely due to the malformation of the longitudinal visceral muscles.

Since the circular muscles were only slightly affected in *rols* mutants, we focused on the development and fusion process of the longitudinal gut muscles by analyzing the differentiation of longitudinal FCs in *rols* mutant embryos carrying the reporter construct *HLH45F-lacZ*[21]. We found that the longitudinal FCs migrated correctly along the circular muscles during mid-embryogenesis and were arranged dorsally and ventrally to them (compare *rols* mutant in Figure 4G with wild-type in Figure 1E). However, shortly before constrictions formed, the cells did not align entirely perpendicular to the circular muscles; instead, they formed protrusions in other directions and were mainly mononucleated (Figure 4C, arrowheads) at a stage when binucleated and trinucleated syncytia were detectable in the wild-type (Figure 1F). At later stages, we observed gaps between the normally evenly distributed cells (compare *rols* mutant in Figure 4H–J to the wild-type in Figure 1I). We sometimes detected mononucleated cells with protrusions stretching in all directions, but rarely detected stretched and binucleated cells in the anterior part of the gut. Interestingly, the posterior part of the gut was still surrounded by dense stripes of longitudinal muscles in late embryogenesis (Figure 4H and J). When we looked at the cells at a higher magnification, we observed mainly binucleated cells with protrusions orientated in the correct anterior–posterior direction (Figure 4D).

In summary, although the longitudinal FCs migrated correctly, fusion of these cells was disturbed in *rols* mutant embryos. As a consequence, only binucleated syncytia were detected at the end of embryogenesis, which could also indicate a delay in their fusion. In the anterior part of the gut, the phenotype was more severe, and the longitudinal muscles mainly comprised mononucleated cells. Notably, longitudinal muscle fusion proceeded in *rols* mutant embryos, but in analogy to somatic muscle fusion, the fusion process may be limited to the first fusion step that gives rise to binucleated longitudinal syncytia.

### Longitudinal muscle development requires Lame duck

The requirement for Rols7 in longitudinal muscle fusion could indicate a broader similarity between myoblast fusion in somatic and visceral longitudinal myogenesis. Therefore, we analyzed longitudinal visceral muscle development and midgut morphology in the background of mutations that disturb somatic muscle fusion at different fusion-relevant steps.

**Figure 5:**
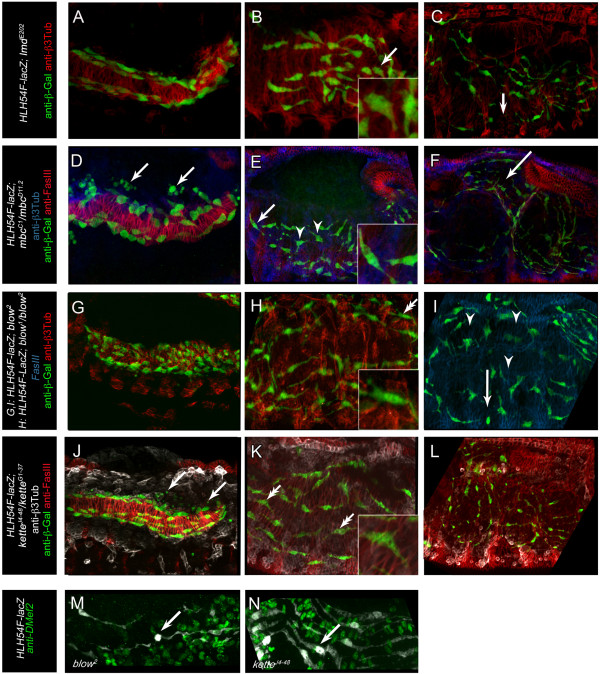
**Longitudinal muscle development is disturbed in *lmd, mbc, blow, *and *kette *mutants. (A–C)***lmd*^*E202*^, **(D–F)***mbc*^*C1*^/*mbc*^*D112*^, **(G–I)***blow*^*2*^ and *blow*^*1*^/*blow*^*2*^, and **(J–L)***ketteJ*^*-48*^/*kette*^*G1–37*^ mutant embryos carrying the reporter construct *HLH54F*-*lacZ* and labeled with anti-β-Gal (green), anti-FasciclinIII (anti-FasIII, red in **D**–**F** and **J**–**L**, blue in **I**) and anti-β3-Tubulin (anti-β3Tub, blue in **D**, **F**, **J**–**L** and red in **A**–**C**, **G**, **H**). **(A)** Unfused longitudinal muscles in an *lmd*^*E202*^ mutant embryo at stage 14. **(B and C)** Properly oriented protrusions (arrows in **B**, inset) and initial midgut chambering (arrow in **C**) in an *lmd*^*E202*^ mutant embryo at late embryogenesis. **(D)** Longitudinal visceral muscle migration in a transheterozygous *mbc*^*C1*^/*mbc*^*D112*^ mutant embryo. Arrows point to aberrantly migrating longitudinal FCs. **(E–F)** Reduction of β-Gal*-*positive cells and abnormal protrusion formation (arrow in **E**, inset) in a transheterozygous *mbc*^*C1*^/*mbc*^*D112*^ mutant embryo at stage 16. Arrow in **(F)** points to region of the midgut not covered by longitudinal FCs. **(G)** Longitudinal FCs migrating all over the circular muscles in a *blow*^*2*^ mutant embryo during mid-embryogenesis. **(H)** Mononucleated longitudinal FCs forming protrusions in random directions (double arrow; inset is a magnification of the area) in a *blow*^*1*^/*blow*^*2*^ embryo. **(I)***blow*^*2*^ embryo showing defects in constriction formation (arrow) and gaps (arrowheads) between the longitudinal cells; compare to less severe phenotype of *blow*^*2*^*/blow*^*1*^ transheterozygous embryo in **(H)**.** (J)** Transheterozygous *kette*^*J4–48*^/*kette*^*G1–37*^ mutant embryo with longitudinal FCs along circular muscles. Some cells were not attached to the circular visceral track (arrows). **(K and L)***kette*^*J4–48*^*/kette*^*G1–37*^ mutant embryo at the end of embryogenesis, with thin cell protrusions (double arrows in **K**; inset is a magnification) of the longitudinal FCs. Stretched, mononucleated longitudinal muscles at the end of embryogenesis in *blow*^*2*^**(M)** and *kette*^*J4–48*^**(N)** mutant embryos. Arrows point to nuclei of longitudinal muscles, marked by anti-DMef2 staining.

Lame duck (Lmd), a homolog of the Gli family of transcription factors in *Drosophila*, is an essential regulator during FCM specification in somatic myogenesis, and thus has an indirect effect on fusion by regulating the expression of *sns*[63]. We previously identified *lmd*^*E202*^ as a new allele of *lame duck* by screening for genes relevant for myogenesis ([19,34]; Holz and Renkawitz-Pohl, unpublished data). An analysis of longitudinal visceral muscle development in homozygous *lmd*^*E202*^ mutant embryos with the *HLH54F-LacZ* reporter and β3-Tubulin antibodies (Figure 5A–C) revealed only mononucleated *lacZ*-positive cells, which indicated that Lame-duck-dependent specified FCMs are also required for longitudinal visceral muscle formation.

In contrast, longitudinal visceral muscle migration and later spreading as well as protrusion formation appeared unaffected in mutant embryos at stages 13 and 14/15 (Figure 5A and B), although morphological defects could be detected within the underlying circular muscle strands. At the end of embryogenesis (Figure 5C), midgut chambering and constriction formation remained incomplete in *lmd*^*E202*^ embryos, reflecting a visceral phenotype as already observed in *sns* mutant embryos [25].

### Longitudinal muscle development requires the F-actin-regulating proteins Myoblast City, Blow, and Kette

Since we found FuRMAS-like structures with actin foci during fusion of longitudinal FCs with FCMs (Figure 2E–H), we analyzed regulators of F-actin polymerization required for somatic myoblast fusion. Mbc is a guanine nucleotide exchange factor (GEF) for the Rac1-GTPase, which is involved in the activation of the Scar/WAVE complex [64]. The loss of Mbc function leads to a block of somatic myoblast fusion, and Mbc accumulates together with Rac1 in actin foci [47]. Furthermore, Mbc is necessary and sufficient in FCMs for myoblast fusion [47]. However, longitudinal visceral muscle migration appeared to be unaffected in transheterozygous *HLH54F-lacZ*; *mbc*^*C11*^/*mbc*^*D11.2*^ embryos (Figure 5D). In contrast to the situation in the wild-type, in the mutant, the longitudinal FCs along the remaining visceral mesoderm were not only still mononucleated at stage 13/14, but also were roundish and had shorter protrusions (Figure 5D), in agreement with data from dye injections into *mbc* mutant muscles [8]. In late embryonic development, the first midgut constriction was missing in *mbc* mutant embryos (Figure 5F). Filamentous protrusions of the longitudinal FCs were then clearly visible at the posterior half of the midgut, while only a few longitudinal FCs with shorter, randomly orientated protrusions were visible in the anterior midgut regions.

Mutations in numerous other members of the Scar/Wave and WASp complexes involved in the Arp2/3-dependent F-actin polymerization machinery induce characteristic fusion defects in the somatic musculature. However, initial studies have only revealed a minor influence of these factors, e.g., Blow and Kette (also referred to as Hem-2 or Nap1), on circular visceral muscle development [10,38]. Therefore, we asked whether these genes are also dispensable for myoblast fusion to form the longitudinal visceral muscles.

While Blow was expressed in the FCMs during fusion and longitudinal myogenesis (Figure 2H–H”), *kette* was broadly transcribed in the embryo until stage 14, and is also maternally contributed [45]. We analyzed whether gut constrictions were correctly formed in *kette* and *blow* mutants. Although the gut chambers were constricted, their proportions differed from that of the wild-type (not shown). We then asked whether the longitudinal muscles develop correctly in *blow* and *kette* mutants. In *HLH54F-lacZ*; *blow*^*2*^ mutants (Figure 5G and I) and in transheterozygous *HLH54F-lacZ*; *blow*^*1*^/*blow*^*2*^ embryos (Figure 5H), longitudinal FCs migrated along the circular mesoderm, although not only dorsally and ventrally, but also along the whole TVM. Moreover, these cells were mononucleated at this time and were rounder than those in wild-type embryos. At later stages, protrusions of the longitudinal FCs stretched not only in anterior–posterior directions, but also in dorsal–ventral directions (Figure 5I).

When we stained *HLH54F-lacZ*; *blow*^*2*^ mutant embryos with an antibody against DMef2, which marks the nuclei of all muscle cells, we observed that cells in the anterior midgut region were mononucleated before the gut became constricted (Figure 5M, arrow). After constriction formation, cell protrusions were much shorter than in the wild-type, and gaps appeared between the neighboring cells (Figure 5I). Although we observed that the phenotype was less severe in the posterior midgut regions, *HLH54F-lacZ-*positive cells remained mainly mononucleated, and only occasionally were binucleated cells found (Figure 5H).

Longitudinal FC migration along the TVM was also observed in transheterozygous *HLH54F-lacZ*; *kette*^*J4–48*^/*kette*^*G1–37*^ embryos, although some *HLH54F-lacZ-*positive cell were localized aberrantly (Figure 5J, arrows). During later stages, longitudinal FCs formed protrusions that were mainly oriented in the correct anterior–posterior direction (Figure 5K, double arrows). At this stage, some *HLH54F-lacZ*-positive myoblasts appeared to be binucleated. However, double staining with anti-DMef2 revealed mainly mononucleated and a few binucleated *HLH54F-lacZ-*positive myoblasts in *kette*^*J4–48*^ mutant embryos at this time (Figure 5N, arrow). At the end of embryogenesis, although the longitudinal FCs had formed thin cell protrusions and aligned along the midgut, overall fewer elongated longitudinal FCs appeared to be present, and some areas along the circular muscles were not covered with longitudinal muscles (Figure 5L).

### Scar/Wave is mainly essential for longitudinal visceral fusion

**Figure 6:**
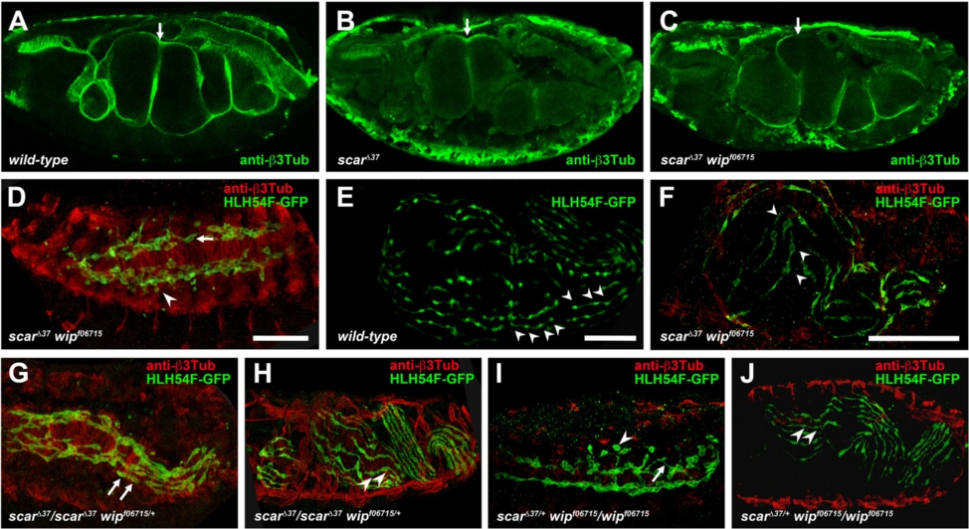
***scar *is required for longitudinal fusion, and FC migration/fusion is promoted by *wip. *(A–C)** Lateral view of stage 16 embryos stained with anti-β3-Tubulin to visualize gut constrictions. **(A)** Wild-type embryo showing normal gut constrictions (arrow). **(B)** Homozygous scar^Δ*37*^ single and **(C)***scar*^Δ*37*^*wip*^*f06715*^ double mutant embryos showing normal gut constrictions, but aberrant gut morphology. **(D, F)** Homozygous *scar*^Δ*37*^*wip*^*f06715*^ double mutant embryos carrying *HLH54F-GFP* to mark longitudinal myogenesis. **(E)** Late stage 15 embryo expressing *HLH54F-lacZ* in a wild-type background. **(D)** Stage 13 embryo showing mononucleated myoblasts (arrow) and myoblasts with migration defects (arrowhead). **(F)** Stage 15 embryo displaying binucleated (two arrowheads) or mononucleated myoblasts (one arrowhead). **(G–J)** Gene dosage experiments. Embryos were stained with anti-β-Gal, anti-β3-Tubulin, and anti-FasIII. **(G, H)** Homozygous *scar*^Δ*37*^ mutant embryo carrying *HLH54F-GFP* and lacking one copy of *wip*^*f06715*^. **(G)** Late stage 13 embryo with normal longitudinal myoblast migration. Sometimes binucleated cells were seen (arrows). **(H)** Stage 16 embryo with binucleated gut muscles (arrowheads). **(I, J)** Homozygous *wip*^*f06715*^ mutant embryo carrying *HLH54F-GFP* and lacking one copy of *scar*^Δ*37*^. **(I)** Stage 13 embryo showing aberrant cell migrations (arrowhead) and abnormal protrusion formation (arrow). **(J)** Stage 15 embryo with binucleated gut muscles (arrows). Scale bars: 50 μm.

Mbc and Kette are both involved in Scar/Wave-dependent activation of the Arp2/3 complex during myoblast fusion [10,31,47]. To determine whether also *scar* is involved in longitudinal FC and visceral FCM fusion, we analyzed midgut constriction formation in *scar*^*Δ37*^ mutant embryos. Gut constriction formation and gut morphology showed no defects in comparison to wild-type embryos (Figure 6A and B). *scar* is also maternally transcribed, and maternal *scar* compensates for the loss of zygotic *scar* in somatic myoblast fusion. Furthermore, *scar* cooperates with WASp-dependent Arp2/3 regulation during somatic myoblast fusion, and in this system fusion is only blocked completely in *scar*^*Δ37*^*wip*^*f06715*^ double mutant embryos [30,36]. Our findings that *blow* mutants also displayed longitudinal visceral fusion defects further imply an involvement of WASp, as recent studies have shown that Blow competes with WASp for Wip-binding [31]. However, *wip* single mutants, *Arp3*^*schwächling*^ single mutants, and *Arp3*^*schwächling*^*wasp* double mutants did not show severe defects in longitudinal visceral muscle formation (Additional file 4: Figure S4B, C and I–I”), which might be due to maternal contribution in the case of *Arp3* and *wasp*. However, at stage 13, some longitudinal FCs showed aberrant cell migration (Additional file 4: Figure S4H, arrow). Nevertheless, at the end of embryogenesis, gut morphology and constrictions almost like those in the wild-type appeared, and at least trinucleated muscles were observed (Additional file 4: Figure S4I–I”). This finding may indicate that WASp-dependent Arp2/3 activation is involved in the migration of longitudinal FCs.

To analyze the fusion defects of *scar*^*Δ37*^*wip*^*f06715*^ double mutants more closely, we examined gut constriction formation (Figure 6C) and introduced the *HLH54F-GFP* marker for longitudinal FCs into *scar*^*Δ37*^*wip*^*f06715*^ double mutants (Figure 6D, F). We observed migrating, mostly mononuclear FCs in stage 13 embryos (Figure 6D, arrow), some of which showed aberrant migration positions (Figure 6D, arrowhead). The fusion defects were severe; we detected mainly mononucleated, binucleated, and occasionally trinucleated *HLH54F-GFP*-positive cells (Figure 6F, arrowheads). Next we asked whether removing a copy of *wip* influences the *scar* phenotype (Figure 6G, H). The FCs at stage 13 were mainly mononucleated during their migration (Figure 6G); we observed fewer FCs at aberrant positions than in embryos with two mutant copies of *wip* (Figure 6D). At stage 16, longitudinal muscles were mononucleated and binucleated (Figure 6H, arrowheads). These muscles showed long extensions and a relatively parallel arrangement in the posterior part of the embryo (Figure 6H). Next we analyzed *wip* homozygous mutants with one mutant copy of *scar*^*Δ37*^ (Figure 6I, J). In the *wip* homozygous embryo, many FCs were at aberrant positions (Figure 6I, arrowhead) and showed aberrant protrusions (Figure 6I, arrow). Muscles at stage 15/16 were mainly mononucleated or binucleated. We concluded that Scar/Wave is an important regulator for longitudinal fusion. The analysis of *scar wip* double mutants further revealed that Wip enhances the defects in longitudinal myogenesis. Because *wip* single mutants did not show any defects, it needs to be clarified whether Wip influences indeed fusion or other processes during longitudinal visceral myogenesis, e.g., migration or stretching.

In conclusion, fusion of the longitudinal FCs with FCMs was severely disturbed in *rols*, *mbc*, *blow, kette,* and *scar* mutant embryos. Our results also revealed significant differences in protrusion formation between these mutants. While cell protrusions frequently formed, but without longitudinal syncytia formation, in *lmd* mutants and in the posterior midgut regions of *mbc* mutant embryos, *blow* and *mbc* mutant embryos had shorter protrusions and abnormal orientation of longitudinal FCs at the anterior midgut. Importantly, the analyses of *scar* mutants indicated that the Arp2/3 activator Scar/Wave but not WASp is mainly essential for the fusion of longitudinal myoblasts and suggested that *blow* may act in this context independently of WASp/Wip-dependent Arp2/3-based actin polymerization.

## Discussion

We analyzed the development of syncytial longitudinal visceral muscles of *Drosophila,* focusing on myoblast fusion. We found that fusion of longitudinal FCs with FCMs depends on several but not all proteins known from the somatic myoblast fusion process.

### FuRMAS-like cell-adhesion structures with F-actin foci form during longitudinal visceral myoblast fusion

At the site of fusion, Duf/Kirre and Rols7 were localized in FCs, and foci of Blow and F-actin-GFP were localized in FCMs. These findings correspond to the FuRMAS structure including the characteristic F-actin-foci observed during somatic myoblast fusion. However, we found distinct differences in the need for and/or function of Scar/Wave and WASp complexes in the two types of myoblast fusion.

### Longitudinal visceral fusion appears to be mainly dependent on Kette and Scar/Wave

The analyses of single and double mutants that possess defects in Arp2/3-induced actin polymerization demonstrated that Scar-dependent Arp2/3 activation is essential for longitudinal myoblast fusion. It has been proposed that the activation of Scar/Wave depends on the small Rac-GTPase [64]. The myoblast-fusion-relevant GEF for Rac is Mbc [47]. Rac involvement is likely required in longitudinal fusion, as longitudinal myoblasts did not fuse in *mbc* mutants. Furthermore, Kette was essential for fusion, in agreement with the function of Kette as part of the Scar/Wave complex. However, the *scar* phenotype was enhanced when also *wip* was deleted. Although, *wip* single mutants did not display longitudinal fusion defects, Wip and its interaction partner WASp might be required for the migration of longitudinal FCs or stretching of longitudinal muscles.

In contrast to *mbc, kette* and *scar*, we detected only minor defects in the Arp2/3 subunit mutant *arp3*^*Schwächling*^. We hypothesized that highly maternally contributed *arp3* mRNA might be sufficient to allow longitudinal fusion. This is in contrast to somatic myoblast fusion [35,36] and axonal pathfinding during CNS development [65]. We further found that gut constrictions were not indicative for loss of longitudinal visceral fusion (Figure 6A–C).

### Blow might act independently of WASp/Wip during longitudinal visceral fusion

During somatic myoblast fusion, Blow is needed to stabilize the WASp/Wip complex, which in turn activates the Arp2/3 complex [31]. Therefore, Blow may act independently of the WASp/Wip complex in the formation of the longitudinal muscles. Furthermore, Blow genetically interacts with Kette during somatic myoblast fusion [34], which indicates a WASp/Wip-independent function of Blow.

### Different relative positions of FCs and FCMs characterize fusion of circular visceral, longitudinal visceral, and somatic muscles

**Figure 7:**
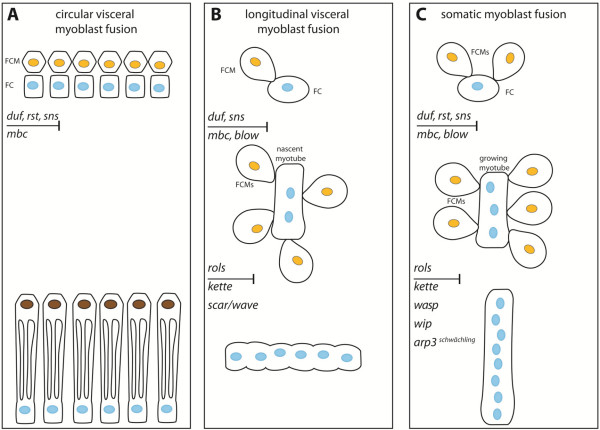
**Model of myoblast fusion creating the circular and longitudinal visceral muscles and the somatic muscles of the *Drosophila *embryo. (A)** Circular visceral muscles arise by incomplete fusion of one FC (blue nuclei) with one FCM (yellow nuclei, after fusion this nucleus is drawn in brown); this depends on Duf, Rst, Sns, and Mbc. **(B)** Longitudinal visceral myoblast fusion leads to syncytia, mostly with six nuclei. Duf (blue nuclei) and Sns (yellow nuclei) are specifically expressed according to cell type during this fusion. In the absence of Mbc*,* no fusion occurs; lack of Rols and Blow leads to a limited number of fusions. **(C)** During somatic myoblast fusion, lack of Duf, Rst, Sns, and Mbc abolishes fusion almost completely; Rols, Kette, WASp, Wip and Arp3^schwächling^ are required for further fusion events to form individual muscles with their characteristic nuclei number.

Differences in the relative position of FCs and FCMs have to be kept in mind when comparing the three different myoblast fusion events in the embryo (Figure 7). The circular muscles originate from a row of FCs fusing with the adjacent row of FCMs, giving rise to a binucleated cell. Thus, fusion takes place in an epithelial-like situation (Figure 7A; only the row of FCMs adjacent to the FCs is shown). However, in longitudinal visceral and somatic myogeneses, FCs and FCMs are not arranged like an epithelium, but rather FCs attract FCMs to the site of fusion [26,27]. Importantly, longitudinal fusion takes place during migration of FCs, which means that defects in migration may mimic a fusion phenotype.

### The complexity of known regulators of myoblast fusion increases from circular to longitudinal to somatic myoblast fusion

Just as in the somatic mesoderm, in the visceral mesoderm, fusion of myoblasts depends on the presence of the immunoglobulin super family receptors Duf/Kirre and/or Rst/IrreC in the FCs, and Sns in the FCMs (Figure 7). In both *duf,rst* double mutants and *sns* mutants, the FCMs fail to adhere to the circular FCs and fusion is completely blocked. Therefore, the adhesion molecules appear to mediate the heterotypic adhesion between these two cell types in all embryonic myoblast fusions [8,11,66,67]. Mutants of the GEF-encoding gene *mbc* displayed mononuclear circular and longitudinal visceral myoblasts as well as somatic myoblasts; thus, also Mbc appears to be essential in all myoblast fusion events.

The first differences in the myoblast fusion events became apparent when we analyzed the adaptor Rols, which is specifically expressed and binds to Duf/Kirre in the FCs. Rols was not essential for the formation of binucleated circular visceral muscles (Figure 7A) but was essential for visceral longitudinal and somatic fusion (Figure 7B, C). We found that Duf/Kirre and Rols localize to the spindle-like FCs during longitudinal visceral muscle myogenesis (Figure 7B), which suggested an interaction as in somatic myogenesis (Figure 7B and C). In *rols* mutants syncytial longitudinal muscle development was disturbed, and we found mostly binucleated mini-muscles. The first fusion events of the longitudinal FCs might function without Rols7, analogous to the first phase of fusion in the somatic mesoderm, which is independent of Rols7 function [40,41,43]. In longitudinal visceral fusion, the adaptor protein Rols7 might bind to Duf/Kirre to allow efficient fusion via a positive feedback loop, as is the case in the somatic mesoderm [68].

Actin foci are typical at the sites of longitudinal visceral and somatic fusion. The Scar/Wave and WASp/Wip complexes bind and activate the Arp2/3 complex and thereby enable actin polymerization during somatic myoblast fusion [30,33,36,37,69]. Our study and other studies showed that actin regulator molecules that perform an essential function during somatic fusion (Figure 7C) play a different role in morphogenesis of the gut muscles, even though they are expressed in the visceral mesoderm. In *blow* and *kette* mutants, stretching and outgrowth of the circular visceral muscles is disturbed, although fusion itself is not affected [10]. This is in contrast to the situation during longitudinal visceral fusion, where Blow, Kette, and Scar/Wave were required (Figure 7A, B). In the case of *kette* and *scar* mutants, it should be considered that a pool of both transcripts is supplied maternally ([45,65]) and that this pool might allow the observed limited number of fusions in homozygous *kette* and *scar* mutants.

Both circular and longitudinal visceral myoblast fusion seemed to be independent of the actin regulators WASp and the WASp-interacting protein Wip (our results) and do not exhibit any obvious defects in gut muscle development [38]. However, WASp and Wip influence the migration of longitudinal FCs.

## Conclusions

Based on our results, we conclude that longitudinal visceral muscles arise by fusion of one FC with several FCMs during FC migration from posterior to anterior locations. The known molecular repertoire needed for longitudinal fusion is more complex than that for circular visceral fusion, but less complex than that for somatic myoblast fusion. Longitudinal myoblast fusion shares cell-adhesion molecules and Mbc with circular visceral and somatic myoblast fusions. Rols acts as an adaptor and likely as a signaling molecule in the FCs and is needed for longitudinal and somatic myoblast fusions, whereas its loss only causes minor defects in visceral circular fusion. Among the actin regulators known from somatic fusion, only Blow, Kette, and Scar/Wave seem to be essential for longitudinal visceral muscle fusion (Figure 7). We hypothesize that this correlates with smaller rings of adhesion molecules and actin foci that do not increase in size during fusion of longitudinal myoblasts owing to the lack of Wip and WASp as regulators of Arp2/3. It is proposed that Blow modulates the stability of the WASp-Wip complex during somatic myoblast fusion [31] and genetically interacts with Kette [34]. As Wip and WASp are not essential for longitudinal fusion, future experiments need to clarify the role of Blow and Kette within the FuRMAS during longitudinal fusion.

We showed that myoblast fusion is not a uniform process, but is characterized by context-dependent modulation. In the future, it needs to be clarified whether these fusion events share a fusogen that leads to membrane fusion and how this event is connected to the so far known differential regulation that prepares myoblasts for membrane fusion.

## Competing interests

The authors declare that they have no competing interests.

## Authors’ contributions

AR carried out the crossings and immunohistochemical analyses of embryos and larval guts, and drafted the manuscript. DB carried out the anti-Duf and anti-Rols7 staining, and coordinated the *rols7* gene regulatory studies. MJ carried out the *rols7* fluorescent *in situ* hybridization. SFÖ contributed conceptionally to the revision of the original manuscript and changed the manuscript accordingly; carried out immunohistochemical analyses of embryos. GW participated in *rols7* fluorescence *in situ* hybridization, initial fly crossings, confocal microscopy analyses of *lmd* mutants, and helped drafting the manuscript. DK carried out *rols7* gene regulation experiments. MP carried out *rols6* gene regulation experiments. SB cloned the *rolsIn1-LacZ* construct and established corresponding transgenic flies. AH conceived the study, participated in its design, carried out initial crossings and initial immunohistochemical analyses of embryos, and confocal microscopy analyses. RR-P supervised and coordinated the work, and helped write the manuscript. All authors read and approved the final manuscript.

## Authors’ information

Anne Holz and Susanne F Önel: equal contributors. Susanne F Önel: corresponding author.

## Supplementary Material

Additional file 1: Figure S1 (A) Midgut isolated from 1^st^ instar larvae expressing the protein-trap fusion protein Sls::GFP were counterstained with TRITC-coupled phalloidin to visualize sarcomeric actin filaments. Arrowheads indicate GFP-positive Z-discs. The sarcomeres of the circular visceral muscles measured at least 10 μm (Figure 2A, arrowheads) in agreement with ultrastructural data [5], while the body wall muscles contain sarcomeres of 1–2 μm in length [70]. (B) Midgut isolated from 1^st^ instar larvae expressing the protein-trap fusion protein Trol::GFP. Trol::GFP localized to the ECM, and the circular visceral muscles seemed to be attached to a layer of Trol-positive ECM when development was completed. Arrows indicate longitudinal muscles, arrowheads point to circular muscles (mainly out of focus), and asterisks mark positions of the nuclei. (C) 1^st^ instar gut muscles of *rp298-lacZ* larvae. Nuclei were counterstained with DAPI; muscles were visualized using an anti-Tropomyosin antibody. Arrowheads indicate nuclei of the circular muscles that were β-Gal negative. Click here for file

Additional file 2: Figure S2 Duf, Rols7, and Blow are expressed in the visceral mesoderm. Wild-type late stage 10 embryos prior to longitudinal visceral fusion labeled with (A and A’) anti-Kirre, (B and C) anti-Rols7, and (D and E) anti-Blow. Arrow in (B) points to the caudal visceral mesoderm, the origin of longitudinal FCs. Arrow in (C) points to Rols7-positive circular FCs of the TVM, and arrowheads point to overlying visceral FCMs devoid of Rols7. Arrow in D points to Blow-expressing visceral mesoderm (for details see [10]) and somatic mesoderm (arrowhead). Arrows in E point to Blow-positives spots in visceral FCMs. Click here for file

Additional file 3: Figure S3 The first intron of *rols7* guides reporter gene expression in longitudinal visceral myoblasts. Reporter gene expression was monitored by anti-β-Gal (A–D) *rols7* reporter lines (E and F) *rols6* reporter lines. (A) Embryo stage 12; *rols7-up3kb-lacZ* (abbreviation: RPL) with 3 kb upstream region was required for strong expression in the somatic mesoderm. (B) Embryo stage 12; *rols7*-*up18bp-lacZ* (abbreviation: RPS) specifically expressed *rol7* in the somatic mesoderm at a low level. (C) Embryo stage 13; *rols7-up3kb-lacZ* (abbreviation: RPL) does not confer expression in the visceral mesoderm (arrow). (D) Embryo stage 13; *rols7In1-lacZ (RolsIn-LacZ)* expressed β-galactosidase in the longitudinal visceral myoblasts only when the intron between exons 1 and 2 of *rols7* was present*.* (E) Embryo stage 14; *rols6-up3kb-lacZ* (abbreviation: R6L) is expressed in the endoderm (arrows) and the primordial for the Malphigian tubules (arrowhead) in agreement with its transcription pattern [43,71]. (F) Embryo stage 16*; rols6-u1.1kb-lacZ* (abbreviation: R6S) showing transcription of *rols6* in the endoderm (arrow) and in Malphigian tubules (arrowhead) only when a 1.2 kb region upstream of the transcription start site is present; no evidence for *rols6* transcription in the mesoderm. Click here for file

Additional file 4: Figure S4 Longitudinal muscle development is not significantly disturbed in *wip* and *arp3* single, and *wasp arp3* double mutants. (A–C) Stage 14/15 embryos labeled with anti-β-Gal to visualize the *HLH54F-lacZ* reporter expression. (A) Wild-type embryo with multinucleated longitudinal muscles (arrowhead). (B) Homozygous *wip*^*D30*^*/wip*^*f06715*^ mutant embryo with 4 to 5-nucleated longitudinal muscles. (C) Longitudinal muscles of *arp3*^*Schwächling*^ mutants display a reduced number of nuclei in longitudinal muscles. (D and D’) Lateral view of *arp3*^*Schwächling*^ mutant embryo labeled with (D, D’) anti-β-Gal (green) and (D’) with anti-FasIII (gray). At stage 16 longitudinal muscles and gut morphology appeared normal in *arp3*^*Schwächling*^ mutant embryos. (E–H) Stage 13 embryos stained with anti-β-Gal (green) to follow longitudinal FCs migration and anti-FasIII (gray) to visualize circular muscles. (E) Wild-type. (F) Homozygous *wip*^*D30*^*/wip*^*f06715*^ (G) *arp3*^*Schwächling*^ and (H) *arp3*^*Schwächling*^*wasp*^*3D3–035*^ mutant embryo with abnormal migrating longitudinal FCs (arrow). (I–I”) Homozygous *arp3*^*Schwächling*^*wasp*^*3D3–035*^ double mutant embryo stained with anti-β3-Tubulin (red), anti-β-Gal (green) and FasIII (gray). (I) At stage 16, *arp3*^*Schwächling*^*wasp*^*3D3–035*^ mutants show many unfused somatic myoblasts. (I’, I”) Longitudinal muscles form *arp3*^*Schwächling*^*wasp*^*3D3–035*^ mutants (I’, arrowheads) and gut morphology appears normal (I”). Scale bars: 50 μm. Click here for file
