# Supplementary figures and images for: Distinct genetic programs guide Drosophila circular and longitudinal visceral myoblast fusion
Source: BMC Cell Biol. 2014 Jul 8;15:27. doi: 10.1186/1471-2121-15-27 (PMC4169254; doi:10.1186/1471-2121-15-27)

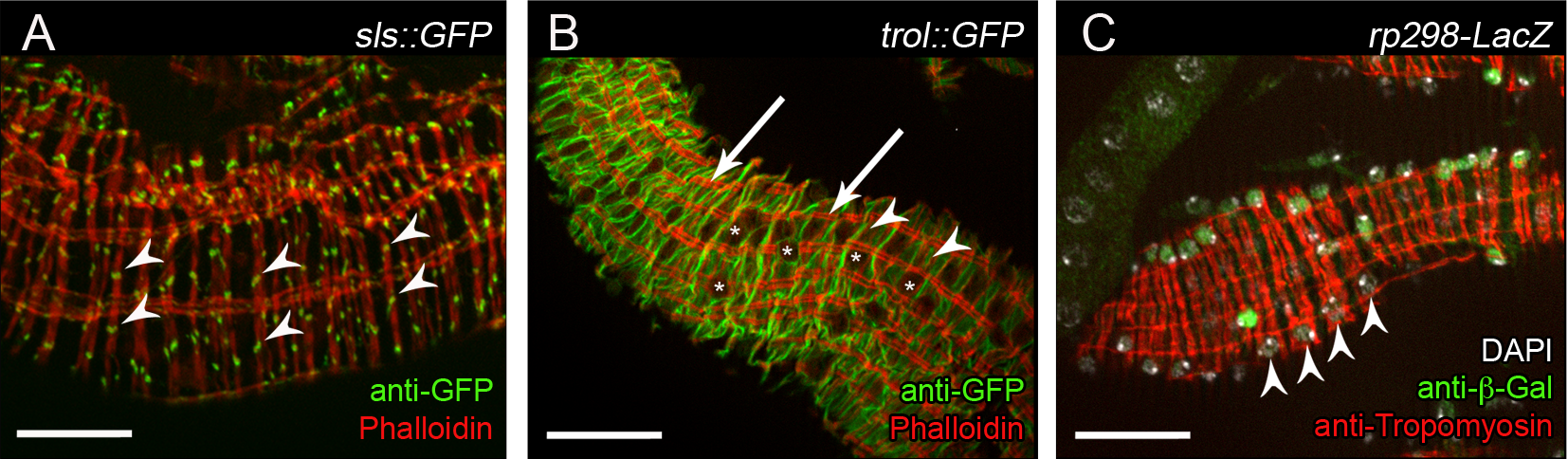

Supplement: Additional file 1: Figure S1 — (A) Midgut isolated from 1st instar larvae expressing the protein-trap fusion protein Sls::GFP were counterstained with TRITC-coupled phalloidin to visualize sarcomeric actin filaments. Arrowheads indicate GFP-positive Z-discs. The sarcomeres of the circular visceral muscles measured at least 10 μm (Figure 2A, arrowheads) in agreement with ultrastructural data [5], while the body wall muscles contain sarcomeres of 1–2 μm in length [70]. (B) Midgut isolated from 1st instar larvae expressing the protein-trap fusion protein Trol::GFP. Trol::GFP localized to the ECM, and the circular visceral muscles seemed to be attached to a layer of Trol-positive ECM when development was completed. Arrows indicate longitudinal muscles, arrowheads point to circular muscles (mainly out of focus), and asterisks mark positions of the nuclei. (C) 1st instar gut muscles of rp298-lacZ larvae. Nuclei were counterstained with DAPI; muscles were visualized using an anti-Tropomyosin antibody. Arrowheads indicate nuclei of the circular muscles that were β-Gal negative. [file 1471-2121-15-27-S1.tiff]

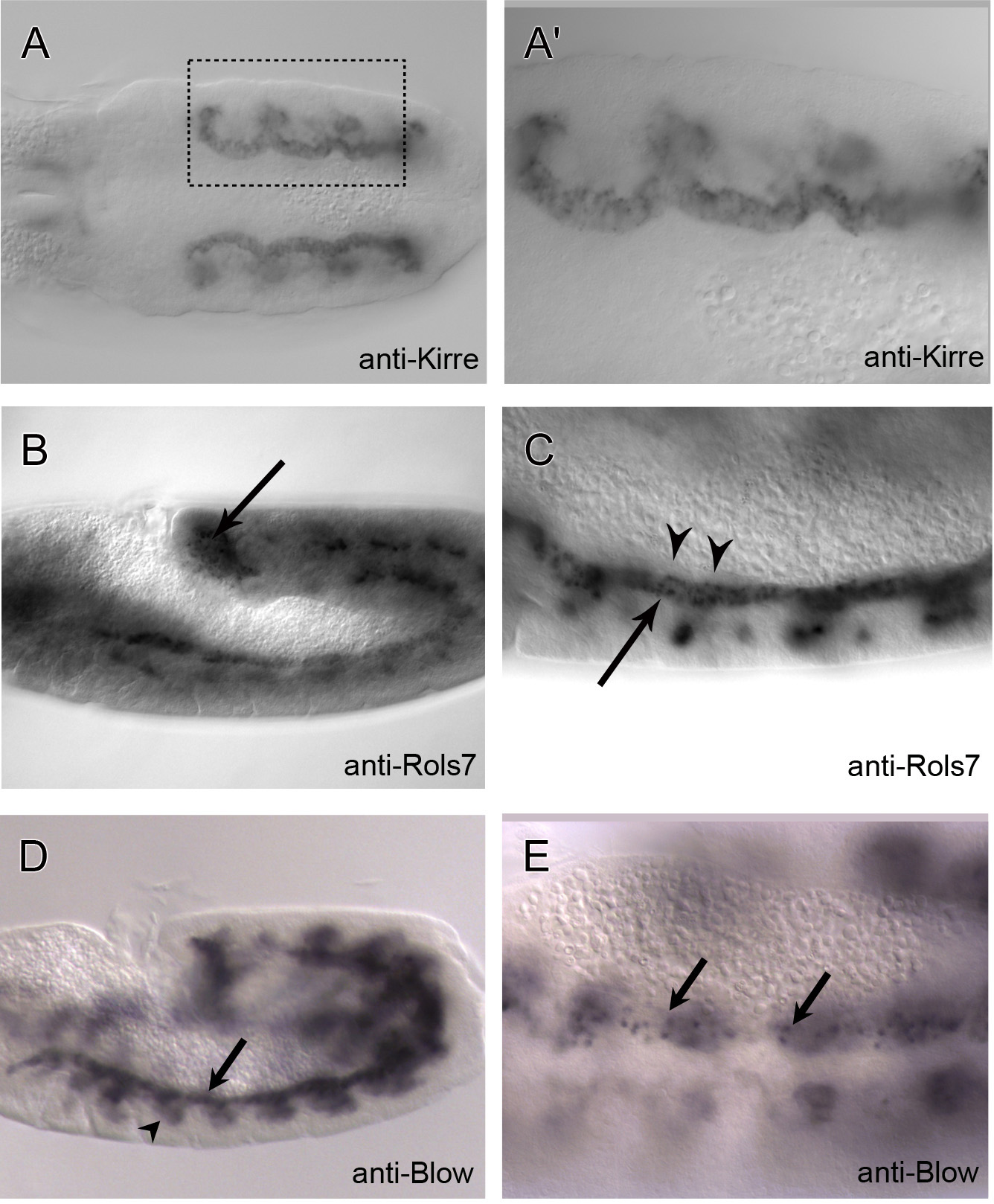

Supplement: Additional file 2: Figure S2 — Duf, Rols7, and Blow are expressed in the visceral mesoderm. Wild-type late stage 10 embryos prior to longitudinal visceral fusion labeled with (A and A’) anti-Kirre, (B and C) anti-Rols7, and (D and E) anti-Blow. Arrow in (B) points to the caudal visceral mesoderm, the origin of longitudinal FCs. Arrow in (C) points to Rols7-positive circular FCs of the TVM, and arrowheads point to overlying visceral FCMs devoid of Rols7. Arrow in D points to Blow-expressing visceral mesoderm (for details see [10]) and somatic mesoderm (arrowhead). Arrows in E point to Blow-positives spots in visceral FCMs. [file 1471-2121-15-27-S2.jpeg]

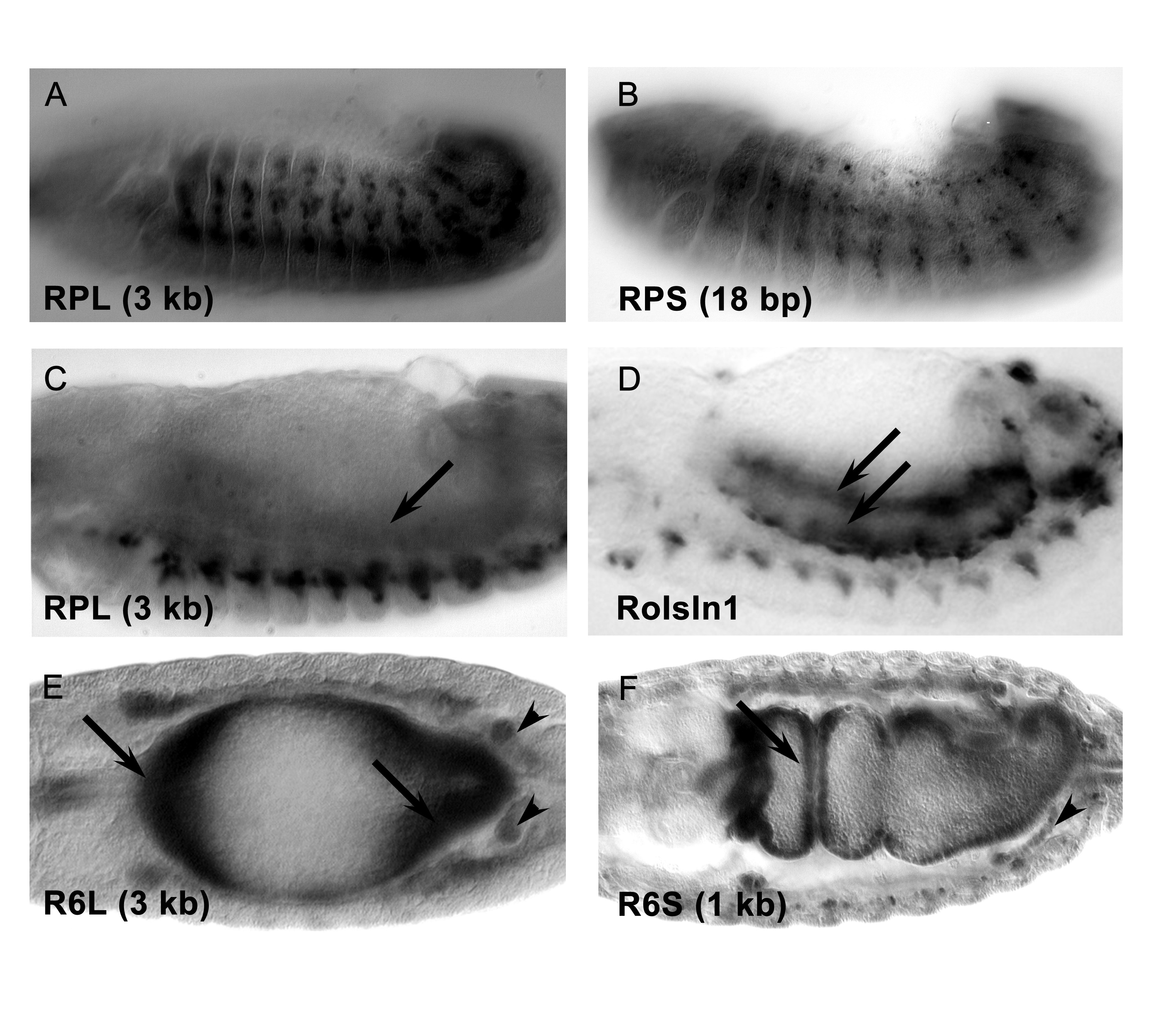

Supplement: Additional file 3: Figure S3 — The first intron of rols7 guides reporter gene expression in longitudinal visceral myoblasts. Reporter gene expression was monitored by anti-β-Gal (A–D) rols7 reporter lines (E and F) rols6 reporter lines. (A) Embryo stage 12; rols7-up3kb-lacZ (abbreviation: RPL) with 3 kb upstream region was required for strong expression in the somatic mesoderm. (B) Embryo stage 12; rols7-up18bp-lacZ (abbreviation: RPS) specifically expressed rol7 in the somatic mesoderm at a low level. (C) Embryo stage 13; rols7-up3kb-lacZ (abbreviation: RPL) does not confer expression in the visceral mesoderm (arrow). (D) Embryo stage 13; rols7In1-lacZ (RolsIn-LacZ) expressed β-galactosidase in the longitudinal visceral myoblasts only when the intron between exons 1 and 2 of rols7 was present. (E) Embryo stage 14; rols6-up3kb-lacZ (abbreviation: R6L) is expressed in the endoderm (arrows) and the primordial for the Malphigian tubules (arrowhead) in agreement with its transcription pattern [43,71]. (F) Embryo stage 16; rols6-u1.1kb-lacZ (abbreviation: R6S) showing transcription of rols6 in the endoderm (arrow) and in Malphigian tubules (arrowhead) only when a 1.2 kb region upstream of the transcription start site is present; no evidence for rols6 transcription in the mesoderm. [file 1471-2121-15-27-S3.jpeg]

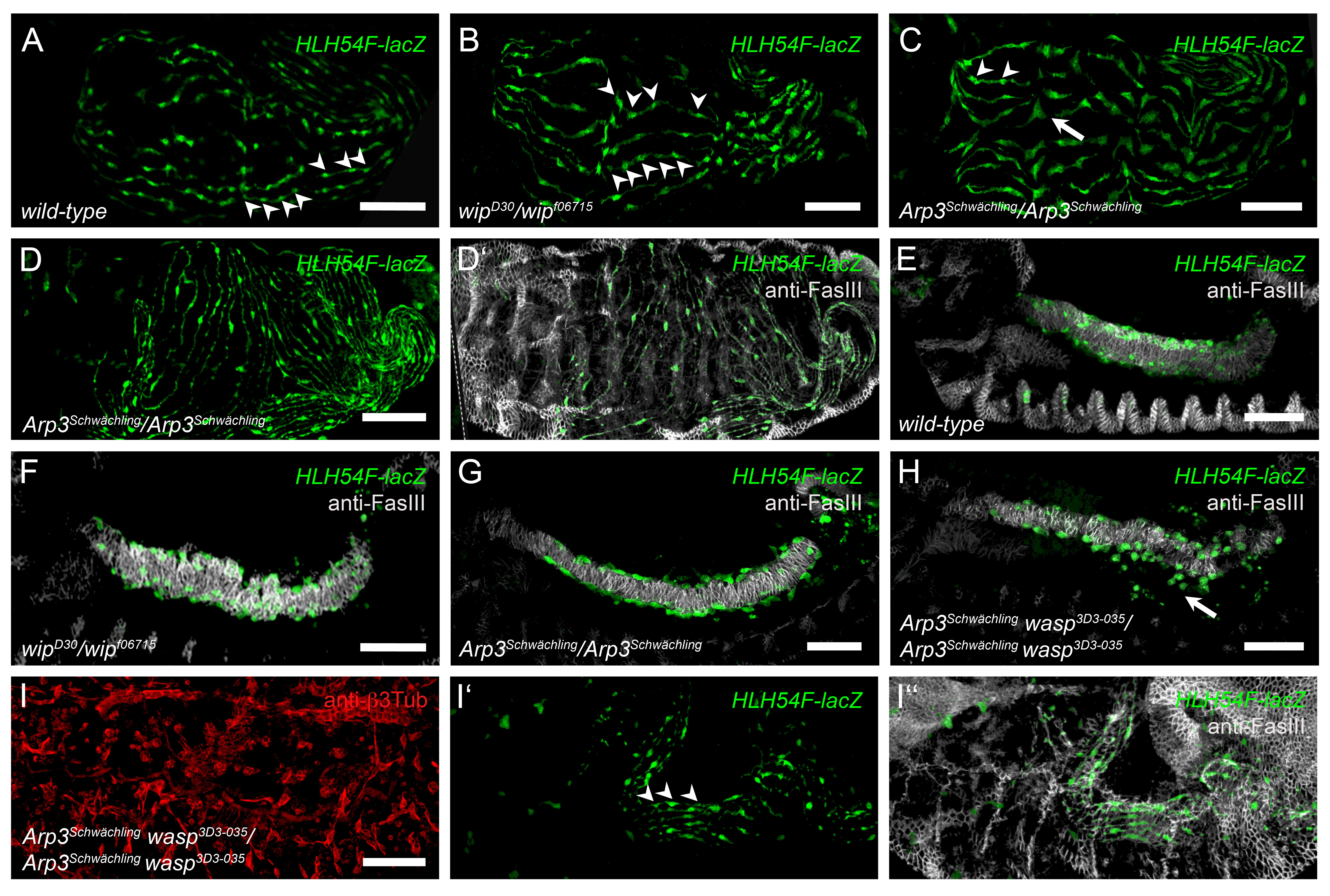

Supplement: Additional file 4: Figure S4 — Longitudinal muscle development is not significantly disturbed in wip and arp3 single, and wasp arp3 double mutants. (A–C) Stage 14/15 embryos labeled with anti-β-Gal to visualize the HLH54F-lacZ reporter expression. (A) Wild-type embryo with multinucleated longitudinal muscles (arrowhead). (B) Homozygous wip D30 /wip f06715 mutant embryo with 4 to 5-nucleated longitudinal muscles. (C) Longitudinal muscles of arp3 Schwächling mutants display a reduced number of nuclei in longitudinal muscles. (D and D’) Lateral view of arp3 Schwächling mutant embryo labeled with (D, D’) anti-β-Gal (green) and (D’) with anti-FasIII (gray). At stage 16 longitudinal muscles and gut morphology appeared normal in arp3 Schwächling mutant embryos. (E–H) Stage 13 embryos stained with anti-β-Gal (green) to follow longitudinal FCs migration and anti-FasIII (gray) to visualize circular muscles. (E) Wild-type. (F) Homozygous wip D30 /wip f06715 (G) arp3 Schwächling and (H) arp3 Schwächling wasp 3D3–035 mutant embryo with abnormal migrating longitudinal FCs (arrow). (I–I”) Homozygous arp3 Schwächling wasp 3D3–035 double mutant embryo stained with anti-β3-Tubulin (red), anti-β-Gal (green) and FasIII (gray). (I) At stage 16, arp3 Schwächling wasp 3D3–035 mutants show many unfused somatic myoblasts. (I’, I”) Longitudinal muscles form arp3 Schwächling wasp 3D3–035 mutants (I’, arrowheads) and gut morphology appears normal (I”). Scale bars: 50 μm. [file 1471-2121-15-27-S4.jpeg]
